# Understanding how the motivational dimension of learning is influenced by clinical teaching in medical education: A prospective cohort study

**DOI:** 10.1016/j.amsu.2021.102366

**Published:** 2021-04-29

**Authors:** Parisa Moll-Khosrawi, Jonathan Steven Cronje, Christian Zöllner, Jens Christian Kubitz, Leonie Schulte-Uentrop

**Affiliations:** a Department of Anaesthesia, University Medical Centre Hamburg-Eppendorf, Martinistr. 52, 20246, Hamburg, Germany; b Department of Anaesthesia, Paracelsus Medical School Nürnberg, Prof.-Ernst-Nathan-Str. 1, 90419, Nürnberg, Germany

**Keywords:** Self-determination theory, Motivation, Medical curricula, Teaching approaches, Simulation-based medical education

## Abstract

**Introduction:**

Many changes of medical curricula have been conducted in the past years. Based on learning psychology, three dimensions of learning have to be covered, in order to create the best possible curricula: Cognitive, metacognitive and motivational. The metacognitive and cognitive dimension (what/how to teach) have always been considered and the motivational dimension has been neglected, although the importance and benefits of motivation in learning have been emphasized repeatedly. One way to influence motivation in medical curricula are the teaching formats, as it has been shown that the construction of a curriculum can influence students’ motivation. So far, evidence about the motivational effects of teaching formats are scarce.

**Methods:**

In a prospective interventional cohort study, 145 3rd year medical students were sampled. The effects of a 3-day bedside teaching in the operating theatre and two simulation-based trainings on students’ motivation (outcome measure) were analysed. It was hypothesized, that the simulation training and the bedside teaching enhance autonomous motivation and decrease controlled motivation.

**Results:**

The bedside-teaching decreased external (controlled) motivation (−0.14, *p* = .013, 95% CI [-0.24, −0.03]), alongside with identified (autonomous) motivation (−0.22, *p* < .001, 95% CI [-0.34, −0.10]). The simulation-based trainings did not change students’ motivation.

**Conclusion:**

To prevent the unintended decrease of identified (autonomous) motivation, undergraduates should be supervised and introduced carefully, when attending bedside teaching in unknown medical fields. Simulation-based medical education certainly has plenty of benefits in medical education but its effects on the motivational dimension of learning needs further investigations.

## Highlights

• Enhancement of medical students' autonomous motivation, we educate future doctors who are intrigued for life-long learning.
• Not only the transmission of knowledge in medical education is important.
• The motivational dimension of learning leads to greater learning success.
• Bedside teaching decreases external motivation but when not introduced well to clinical work places, it also decreases autonomous motivation.
• Simulation-based medical education does not necessarily enhance students' motivation

## 1 Introduction

Medical education and curriculum developments always strive to produce the best learning environment for medical students, pursuing the goal of imparting knowledge and skills, to educate good future doctors [1,2]. These objectives led to many curriculum changes in the past years, [1], which were based on content concerns and dealt with transmission and procession of knowledge [3,4]. However, the importance of the affective (motivational) dimension of learning [5] has been undervalued by medical curriculum developers, [6], even though it has been emphasized that students’ motivation might have a greater impact on individual outcome than learning and teaching strategies [7].

Therefore, the motivational dimension of learning should be considered in all fields of medical education. The commonly applied motivational theory is the “self Determination Theory“ (SDT), described by Deci and Ryan [8,9], which postulates that each human has an innate will to grow and this growth takes place when three basic psychological needs (competence, autonomy, relatedness) are satisfied [10]. Depending on the satisfaction of the basic psychological needs, different types of motivation underlie human behaviour [11]. These types of motivation are described based on a motivational continuum, with intrinsic motivation on the one- and amotivation on the other end. In-between lies extrinsic motivation (external sources form the reason to pursue an activity), which is subdivided in four different types of regulations: external, introjected, identified and integrated [10].

Intrinsic, integrated and identified regulation are summarized as “autonomous self-regulation/motivation” and external and introjected regulation are summarized as “controlled self-regulation/motivation“.

In the past 10 years, medical education researchers have shown that not only intrinsic motivation, but also autonomous motivation, lead to several benefits, amongst them, better learning, better academic achievement, better well-being, perseverance and enthusiasm [7,[12], [13], [14]].

Therefore, enhancing autonomous motivation and decreasing controlled motivation should be a decisive goal in medical education. For this purpose, teaching approaches are reasonable means, because it is known that the construction of a curriculum can influence student motivation [15]. However, knowledge and evidence about motivational effects of teaching formats of a curriculum are scarce [16]. Thus, motivation-enhancing teaching formats should be identified and integrated into medical curricula, then we will be more successful to educate doctors who are intrigued by and interested in medicine and lifelong learning [17].

Based on theoretical assumptions of the SDT, simulation-based medical education (SBME) and bedside teaching, have the potential to stimulate motivation of medical students. Therefore, we investigated in this prospective cohort study, the effects of these two teaching formats on medical students’ situational motivation. Primary outcome was the change of motivation by each teaching format (within each assessment setting) and the secondary outcome was the comparison of the motivational effects of the both teaching formats (between the assessment settings).

We hypothesized that SBME and bedside-teaching enhance autonomous motivation and decrease controlled motivation.

## 2 Methods

Our study is reported in accordance to the STROCCS (strengthening the reporting of cohort studies in surgery) criteria [18].

### 2.1 Registration and ethics

The study is registered in accordance with the declaration of Helsinki in a publicly accessible research registry (ISRCTN, ID: ISRCTN89146039) http://www.isrctn.com/ISRCTN89146039).

We contacted the local Ethics Committee of Hamburg, Germany with a detailed project description and the head of the committee did not see any necessity of deliberation and classified the project as not inevitable for ethic consultation and approved the study. All methods were performed in accordance with the relevant guidelines and regulations (Declaration of Helsinki).

Participation in the study was voluntary and written informed consent was obtained from each study participant.

### 2.2 Protocol and patient involvement

A detailed a priori study protocol was compiled by the research group. This protocol included the primary and secondary outcomes and hypothesis. Furthermore, the certain points of the assessment of outcome measures were stated, as well as the inclusion and exclusion criteria for the study participants.

No study participants were involved in the study design, organisation or data collection.

### 2.3 Study design

We used a prospective interventional cohort study design, which is appropriate to assess the study outcomes.

Motivation and motivational changes were assessed in a cohort of 3rd year undergraduates during a two-week anaesthesiology teaching module. This module was composed of a total of six teaching units, which were attended in the same chronological order. The module started with a 3-h lecture, in which basic learning objectives of anaesthesia were imparted and an introductory to clinical bedside teaching (clerkship) was given. Following this, all undergraduates participated in a 3-day bedside teaching in the operating theatre (intervention for assessment one). Two weeks later, the same undergraduates participated in two simulation trainings which were scheduled within one week (intervention for assessment two).

We assessed undergraduates’ motivation to participate in bedside-teaching and in the short-time repetitive simulation-based emergency trainings.

### 2.4 Study setting

We performed this study at the Department of Anaesthesia of the University Medical Center Hamburg-Eppendorf, during the Winter semester 2018/19. We performed a systematic sampling and recruited two weeks prior to the start of the semester, the 3rd year medical students (*N* = 145) who were assigned to participate in the mandatory 2-week anaesthesia teaching module during the study period.

### 2.5 Cohort groups

The study protocol specified one cohort for the assessment of study outcomes and interventions. Prior to the study, all students were divided into smaller groups by the deanery and based on their schedule, attended the teaching units of the module at different times of the semester. Each subgroup of the cohort participated in the same chronological order in the same teaching units (intervention) and no further subgroup analysis was conducted.

### 2.6 Participants

3rd year students were chosen, because they were already familiar with SBME trainings and bedside-teaching. Eligibility criteria were the familiarity with each teaching format (SBME and seminar), in order to ensure that motivational expressions and changes were not affected and biased by cognitive overload due to unfamiliarity with the teaching formats. Next to familiarity with the teaching formats, no further eligibility criteria were defined.

### 2.7 Recruitment

Two weeks prior to the semester (study period), an email with information about the study was send to all the 3rd year students. A reminder email was send out three days prior to each study week for each student group. Participation in the study was voluntary and written informed consent was obtained from each study participant.

### 2.8 Sample size

The sample size was determined by the size of the 3rd year medical students (systematic sampling). Therefore, no specific calculations regarding the study size were made.

### 2.9 Intervention and considerations

Each teaching unit was defined as an intervention. The changes of motivation after participation in each teaching format (within each assessment setting) was set as the primary endpoint and the comparison of the motivational effects of the both teaching formats (between the assessment settings) was set as the secondary endpoint. The whole cohort of study participants had the same schedule of classes between the interventions, so that concurrent or influencing (motivation) variables were ruled out. The teaching units were conducted by medical educators of the Department of anaesthesiology. In order to reflect real life effects of the teaching formats, they were not changed compared to a non-study setting.

No further post-intervention considerations and follow-up measures were included in the study design.

**Fig. 1:**
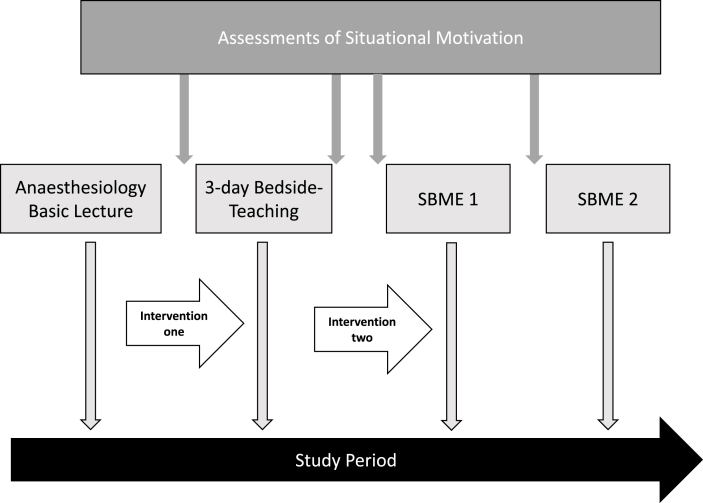
Study design and points of assessments of situational motivation of the 3rd year undergraduates. *Note*: SBME: Simulation-based medical education.

A flowchart of the study procedures and the study design is presented in Fig. 1.

#### 2.9.1 Quality control and operator details

The learning objectives for the bedside teaching were enclosed to the undergraduates as well to all the anaesthesiologists who were involved in the teaching, on a digital platform of the medical faculty of the University of Hamburg. The anaesthesiologists were all familiar with the concept of the bedside teaching and most of them had completed mandatory faculty development sessions on medical education.

#### 2.9.2 Bedside teaching in the operating theatre

Each student was assigned to one anaesthesiologist to accompany him/her during the bedside-teaching, to see and participate in his/her daily work. Thus, the undergraduates were actively involved in the perioperative care of the patients and became familiar with the role of anaesthesia in this context. Furthermore, they had the opportunity to practise technical skills.

Potential bias was ruled out for the SBME, as the students were familiar with the required technical skills and theoretical knowledge, as well as with the concept of simulation training. Hereby we ruled out eventual emotional stress due to unfamiliarity with simulation, technical skills or theoretical knowledge. After each simulation scenario a systematic debriefing took place, providing feedback and emotional support [19]. This debriefing had no time-limit to ensure that the undergraduates were provided with all the answers to their questions.

#### 2.9.3 Repetitive simulation-based emergency training

The two simulation trainings were thematically coherent and mostly dealt with cardiothoracic emergencies and cardiac arrest during anaesthesia.

The trainings were conducted in the simulation center of the department, in which several high fidelity simulators (Resusci Anne, Laerdal Medical AS, Stavanger, Norway) were used. A maximum of 15 students per training participated. Every training had a pre-defined set of standardised scenarios. The students were randomly divided into small groups to conduct the scenario (three students per scenario) in different rooms of the simulation center. Each small group was supervised by one anaesthesiologist, experienced in medical education.

### 2.10 Outcomes

Situational motivation was assessed (paper based) prior and after the bedside teaching (intervention one) and prior and after (= prior to the second SBME training) the first SBME training.

Students were asked to specify the degree to which each item represents a reason for them to participate in the anaesthesia teaching units or in to engage in anaesthesia related topics.

#### 2.10.1 Situational motivation

The translated (German) version of the validated Situational Motivation Scale (SIMS) was used to assess motivation [20,21]. The SIMS measures the situational motivation to partake in a defined task/activity at a specific point of time, using five sub-scales, with four items per subscale resulting in a total of 20 questions/items.

Each item has a 7-point Likert scale (1 = ‘‘Does not correspond at all’’ and 7 = ‘‘Corresponds exactly’’).

**Fig. 2:**
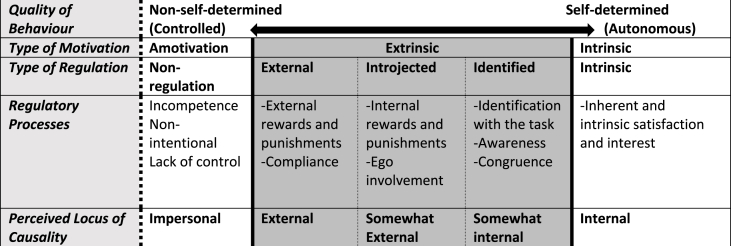
Overview about the motivational levels and regulatory processes described by the SDT (based on Ten Cate et al., 2011 [7]).

An overview of the different types of motivation which are measured by the SIMS with their underlying quality of behaviour is depicted in Fig. 2.

### 2.11 Statistical analysis

We performed statistical analysis with StataCorp. 2017. *Stata Statistical Software: Release 15*. College Station, TX: StataCorp LLC.

Sample characteristics are given as absolute and relative frequencies or mean ± standard deviation, whichever is appropriate.

The changes in the motivation scales caused by the interventions (assessment settings) were analysed separately for each scale. This was done by using a multilevel model with the pre intervention motivation as baseline and the change between pre and post as outcome. The multilevel approach takes into account an additional level for “undergraduate”, as undergraduates received two assessment settings (bedside teaching, SBME), which were not independent from each other. Additionally, an intervention indicator was included in the model as a fixed effect. This approach allows the simultaneous estimation of the between and the within differences of the assessment settings (bedside teaching and SBME) in one model. All of the models present case analyses.

The results were reported as estimated means, which are represented in graphs with their corresponding 95% confidence intervals [95% CIs].

A two-tailed *p* < .05 was considered to be statistically significant. Nominal *p*-values are reported without correction for multiplicity.

To validate the SIMS, we calculated Cronbach's alpha for each item based on the reports from all the settings. This analysis was conducted with IBM SPSS Statistics Version 23.0.

## 3 Results

### 3.1 Participants and questionnaires

All the (*N* = 145) 3rd year undergraduates (male n = 68, age *M* = 22.1, *SD* = 0.84; female n = 77; age *M* = 22.7, *SD* = 1.02) participated within the study. Twenty-six SIMS questionnaires had to be excluded from the second assessment (post-intervention one), ten from the third assessment (pre-intervention two) and twenty-two from the forth assessment (post-intervention two), because they were incomplete or boxes of the questionnaire were not marked clearly. Only questionnaires were included in the final analysis (changes from the baseline) which had corresponding pre- and post- SIMS assessments (n = 138 students; 207 assessments for each motivation level). The baseline of each motivational level was calculated based on all reports (Table 1).

**Table 1:** Motivational changes after each teaching intervention.

	Bedside Teaching						SBME						Cronbach's Alpha *Pre vs. Post for both settings*
	Pre *M SD*	Post *M SD*	Estimated Difference	*p*	95% CI		Pre *M SD*	Post *M SD*	Estimated Difference	*p*	95% CI		
					***LL***	***UL***					***LL***	***UL***	
***Number included for Baseline*** ***Numer included for estimated difference***	145 113	119 113					135 113	113 113					
**Intrinsic**	5.85 .72	5.86 .82	.02	.737	-.07	.11	5.81 .78	5.90 .82	.06	.207	-.03	.15	.84
**Identified**	5.51 1.00	5.25 1.21	-.22	<.001	-.34	-.10	5.31 1.19	5.41 1.17	-.07	.294	-.19	.06	.83
**Introjected**	2.77 1.22	2.78 1.34	.01	.889	-.13	.15	2.82 1.31	2.88 1.37	-.05	.543	-.19	.10	.82
**External**	1.84 .88	1.64 .79	-.14	.013	-.24	-.03	1.80 .81	1.84 .96	-.01	.901	-.12	.11	.83
**Amotivation**	1.51 .77	1.48 .78	.01	.865	-.08	.09	1.45 .74	1.41 .61	-.06	.223	-.15	.03	.84

*Note:* Repetitive measurements with two interventions with non-equidistant washout phase. Pre.: Pre-intervention. Post = Post-intervention. SBME: Simulation-based medical education; CI = confidence interval.

### 3.2 Primary outcome: Changes of motivation after each teaching unit

Overall, the undergraduates reported high levels of autonomous motivation (intrinsic and identified regulation) and low levels of controlled motivation (introjected and external) as well as amotivation at all measurements (Table 1).

The intervention of repetitive simulation had no significant effect on the different motivational qualities, whereas the bedside teaching lead to a significant decrease of students’ external (−0.14; *p* = .013; 95% CI: [-0.24, −0.03]) - and identified motivation (−0.22; *p* < .001; 95% CI: [-0.34, −0.10]) (Table 1). These results indicate, that the bedside-teaching decreased both autonomous self-regulation and controlled self-regulation of the undergraduates.

**Fig. 3:**
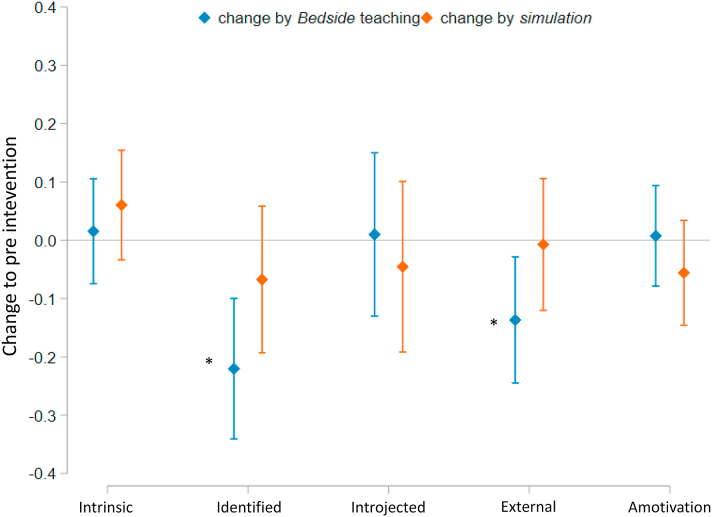
Mean changes of motivation after each teaching intervention in relation to the zero-baseline (set by pre-intervention).

These relative changes of each motivational quality in relation to the zero-baseline (set by the results of SIMS prior to the interventions), are shown in Fig. 3.

The validity calculation (Cronbach's alpha) showed, that the SIMS questionnaire is a reliable and valid tool to assess situational motivation of undergraduates in the context of medical education (Table 2).

**Table 2:** Comparison between settings.

Type of motivation (regulation)		Estimate difference	p	95% CI	
	n			LL	UL
Intrinsic	113	.05	.474	-.08	.17
Identified	113	.15	.074	-.01	.32
Introjected	113	-.06	.592	-.26	.15
External	113	.13	.090	-.02	.28
Amotivation	113	-.06	.283	-.18	.05

Note: CI = confidence interval; LL = lower limit; UL = upper limit; est. = estimated.

### 3.3 Secondary outcome: Comparison of motivational effects of bedside teaching and SBME

Analysis and comparison of the motivational changes (between the two assessment settings) caused by each teaching format showed no significant differences (Table 2). These results indicate, that none of the teaching formats had a stronger effect on students’ motivation than the other.

## 4 Discussion

In our prospective cohort study, we found that a repetitive simulation training (SBME) had no significant effect on situational motivation, whereas a three-day bedside teaching decreased the external and identified motivation of the investigated group of 3rd year medical students.

To our best knowledge, no published investigation has been conducted, analysing if teaching formats could influence student motivation [17,22]. Only as a byproduct- and not mainly focusing on motivational aspects-studies exploring Problem based learning (PBL) found, that students liked more the PBL learning approach and due to their high autonomy in PBL, students showed high levels of intrinsic motivation for PBL and even higher levels for blended PBL [16,[23], [24], [25], [26]].

Translating the manifold study findings conducted based on the SDT, SBME has the potential to enhance autonomous motivation by satisfying the three basic psychological needs (autonomy, competence, relatedness): SBME provides a safe and supportive environment for practising skills [27] and hereby enhances the feeling of self-efficacy and competence [28]. Through feedback, reflection and the feeling of responsibility for patients (even if in an artificial environment), [29,30], autonomy is supported [[31], [32], [33]]. The feeling of relatedness is satisfied by acting in a team together during the simulations scenario [30]. This all leads to enhanced autonomous motivation and automatically the locus of causality (the “why”) for task engagement relocates towards the inside. In other words, the reason to engage in a task or activity is seen from the inside (personal endorsement) and not forced on from external. This process of relocation towards the inside is fostered, when the psychological needs of an individual (relatedness, autonomy, competence) are satisfied [34]. However, although we invested maximal effort to exploit the aforementioned benefits of SBME and made sure that the students were familiar with the concept as well as with the required technical and theoretical skills, we could not prove the predicted benefits of SBME on student motivation. Either our SBME trainings might still not be autonomy supportive enough, or the SBME trainings overstrained the undergraduates.

Interestingly, our results show that the bedside teaching decreased students’ external regulation (feeling that engagement is forced from external) and at the same time decreased students’ identified regulation (identification with the task). The first effect (external regulation) is desirable but the second effect is not. Therefore, at first sight, the results seem contradictory. However, this assumption can be ruled out, because according to SDT different levels of different motivational qualities can be present at the same time. It is important to explain and identify why the bedside teaching decreased identified motivation, then implications for the conduction of this teaching format can be outlined [8]. The decrease of identified motivation derived from the circumstance, that the undergraduates were not familiar with the work-place, which is often the case in bedside teachings. They were directly involved in the work flow, which resulted in great external load [35] and diminished the feeling of competence. This led to decreased identification with the task and therefore reduced levels of in identified regulation [10]. To circumvent this unfamiliarity with medical work places and prevent the high cognitive load, [36], it can be considered that, before undergraduates participate in bedside teachings with real patients, they first attend preparatory simulation-based trainings in which bedside skills are practised. This graded approach to skills, would prevent the diminishment of the feeling of competence and the decrease of identified motivation could even be converted to an increase.

The results of our study (analysis of differences between the two assessment settings) do not favour each of the invested teaching formats, regarding motivational effects on students. We did not find any differences in motivational changes caused by each of the teaching formats. Considering the motivational dimension of learning, both teaching formats are valuable components of medical curricula. Bedside teaching connects learning contents and the theoretical knowledge with a real-life setting, this in turn creates a sense of choice for the students to acquire (anaesthesia) knowledge [8,37]. The effect on student motivation would be-like we showed in our results-a decrease of extrinsic motivation. SBME provides a safe learning space but further clarification studies are needed to identify which “ingredients” of SBME might enhance motivation in medical students.

Some limitations of our study merit consideration. We investigated student motivation in a cohort of 3rd year undergraduates and therefore the transfer of our results to other years of medical school might be difficult. Nevertheless, we assessed the situational motivation, which is detached from the contextual level of motivation, [14], in a cohort of medical students which were familiar with the teaching formats. Hereby, we ensured that students did not experience a high cognitive load which might have biased their motivation due to overstrain [36].

The assessment (SIMS) was repeated four times, which may have led some undergraduates to not filling it out carefully. Nevertheless, the undergraduates were aware that their answers were important and were asked only to return the SIMS, if they were capable to fill it out with diligence. Furthermore, the assessment of situational motivation, which is described in SDT, allows the comparison of different and repeated measurements [38,39]. That means that motivation to engage in a specific activity at an exact time point can be measured situational [40].

Our study has a single center design, however, giving the importance of the motivational dimension of learning and the lack of evidence about teaching approaches and their effects on students’ motivation [16,17], our study provides a first step towards adapting our teaching approaches to the affectional dimension of learning and hereby creating truly learner-oriented teaching. The bedside teachings were conducted by several anaesthesiologists of the department and therefore one can argue that they were not standardised enough. Nevertheless, the teaching contents were enclosed to the educators and in medical education, there will always be a bias based on the personal characteristics of the educator. Furthermore, our results provided on the bedside teaching are detached from the educator and focus on the teaching format and contents.

Although our results arise from anaesthesia teachings, the key message can be transferred to any medical educational field, as bedside teaching is a core instructional design worldwide. We recommend the preparation of undergraduates before entering clinical workplaces to prevent unintended effects on motivation and to foster the actual learning process.

## 5 Conclusion and further research

By providing real-life experiences through bedside-teachings, students’ extrinsic motivation can be decreased and the locus of causality relocates to the inside. Bedside teachings may also decrease identified regulation, therefore, to prevent unintended effects on their motivation, undergraduates should be supervised and introduced carefully when attending bedside teaching in unknown medical fields and a preceding simulation training of the bedside skills should be considered. The effects of SBME on the motivational dimension of learning needs further investigations. Different curriculum designs and components should be investigated with regard to their effects on the motivational dimension of learning and motivation should be acknowledged in future research as an important dependent variable of medical education.

## Funding

This study was not supported by any funding.

## Provenance and peer review

Not commissioned, externally peer-reviewed.

## Ethical approval

We contacted the local Ethic Committee of Hamburg (Ethikkomission der Ärztekammer Hamburg) with a detailed project description and the head of the committee did not see any necessity of deliberation and classified the project as not inevitable for ethic consultation (§ 9 des hamburgischen Kammergesetzes) and approved the study. All methods were performed in accordance with the relevant guidelines and regulations (Declaration of Helsinki).

## Author contribution

PMK has made substantial contributions to the conception and design of the work; as well as the acquisition analysis and interpretation of data; She has drafted the work and substantively revised it. She approved the submitted version (and any substantially modified version that involves the author's contribution to the study) and has agreed both to be personally accountable for the author's own contributions and to ensure that questions related to the accuracy or integrity of any part of the work, even ones in which she was not personally involved, are appropriately investigated, resolved, and the resolution documented in the literature.

JS-C has made substantial contributions to the design of the work and the acquisition of data; he has drafted the work. He approved the submitted version (and any substantially modified version that involves the author's contribution to the study) and has agreed both to be personally accountable for the author's own contributions and to ensure that questions related to the accuracy or integrity of any part of the work, even ones in which he was not personally involved, are appropriately investigated, resolved, and the resolution documented in the literature.

CZ has made substantial contributions to the conception of the work and the interpretation of data; He has substantively revised the work. He approved the submitted version (and any substantially modified version that involves the author's contribution to the study) and has agreed both to be personally accountable for the author's own contributions and to ensure that questions related to the accuracy or integrity of any part of the work, even ones in which he was not personally involved, are appropriately investigated, resolved, and the resolution documented in the literature.

JC-K has made substantial contributions to the conception of the work and the interpretation of data; He has substantively revised the work. He approved the submitted version (and any substantially modified version that involves the author's contribution to the study) and has agreed both to be personally accountable for the author's own contributions and to ensure that questions related to the accuracy or integrity of any part of the work, even ones in which he was not personally involved, are appropriately investigated, resolved, and the resolution documented in the literature.

LSU has made substantial contributions to the conception and design of the work; as well as the acquisition analysis and interpretation of data; She has drafted the work and substantively revised it She approved the submitted version (and any substantially modified version that involves the author's contribution to the study) and has agreed both to be personally accountable for the author's own contributions and to ensure that questions related to the accuracy or integrity of any part of the work, even ones in which she was not personally involved, are appropriately investigated, resolved, and the resolution documented in the literature.

## Consent

Written informed consent was obtained from all the undergraduates.

## Registration of research studies

ISRCTN registry with study ID ISRCTN89146039

## Guarantor

Parisa Moll-Khosrawi

Leonie Schulte-Uentrop

## Declaration of competing interest

None.
